# Repolarizing neutrophils via MnO_2_ nanoparticle-activated STING pathway enhances Salmonella-mediated tumor immunotherapy

**DOI:** 10.1186/s12951-024-02726-8

**Published:** 2024-07-27

**Authors:** Shan Lu, Ze Mi, Peng Liu, Jinsong Ding, Yiran Ma, Jieru Yang, Pengfei Rong, Wenhu Zhou

**Affiliations:** 1 Xiangya School of Pharmaceutical Sciences, Central South University, Changsha, Hunan 410013 China; 2 Department of Radiology, The Third Xiangya Hospital, Central South University, Changsha, Hunan 410013 China; 3 National Clinical Research Center for Geriatric Disorders (Xiangya), Changsha, Hunan 410008 China; 4 Key Laboratory of Biological Nanotechnology, NHC. No. 87 Xiangya Road, Changsha, Hunan 410008 China; 5 Hunan Prize Life Science Research Institute Co., LTD, 229 Guyuan Road, Changsha, Hunan 410008 China

**Keywords:** Tumor therapy, Nanomedicine, Microbial therapy, Immunotherapy, Metal oxides

## Abstract

Engineered *Salmonella* has emerged as a promising microbial immunotherapy against tumors; however, its clinical effectiveness has encountered limitations. In our investigation, we unveil a non-dose-dependent type of behavior regarding *Salmonella*’s therapeutic impact and reveal the regulatory role of neutrophils in diminishing the efficacy of this. While *Salmonella* colonization within tumors recruits a substantial neutrophil population, these neutrophils predominantly polarize into the pro-tumor N2 phenotype, elevating PD-L1 expression and fostering an immunosuppressive milieu within the tumor microenvironment. In order to bypass this challenge, we introduce MnO_2_ nanoparticles engineered to activate the STING pathway. Harnessing the STING pathway to stimulate IFN-β secretion prompts a shift in neutrophil polarization from the N2 to the N1 phenotype. This strategic repolarization remodels the tumor immune microenvironment, making the infiltration and activation of CD8^+^ T cells possible. Through these orchestrated mechanisms, the combined employment of *Salmonella* and MnO_2_ attains the synergistic enhancement of anti-tumor efficacy, achieving the complete inhibition of tumor growth within 20 days and an impressive 80% survival rate within 40 days, with no discernible signs of significant adverse effects. Our study not only unveils the crucial in vivo constraints obstructing microbial immune therapy but also sets out an innovative strategy to augment its efficacy. These findings pave the way for advancements in cell-based immunotherapy centered on leveraging the potential of neutrophils.

**Graphical Abstract:**

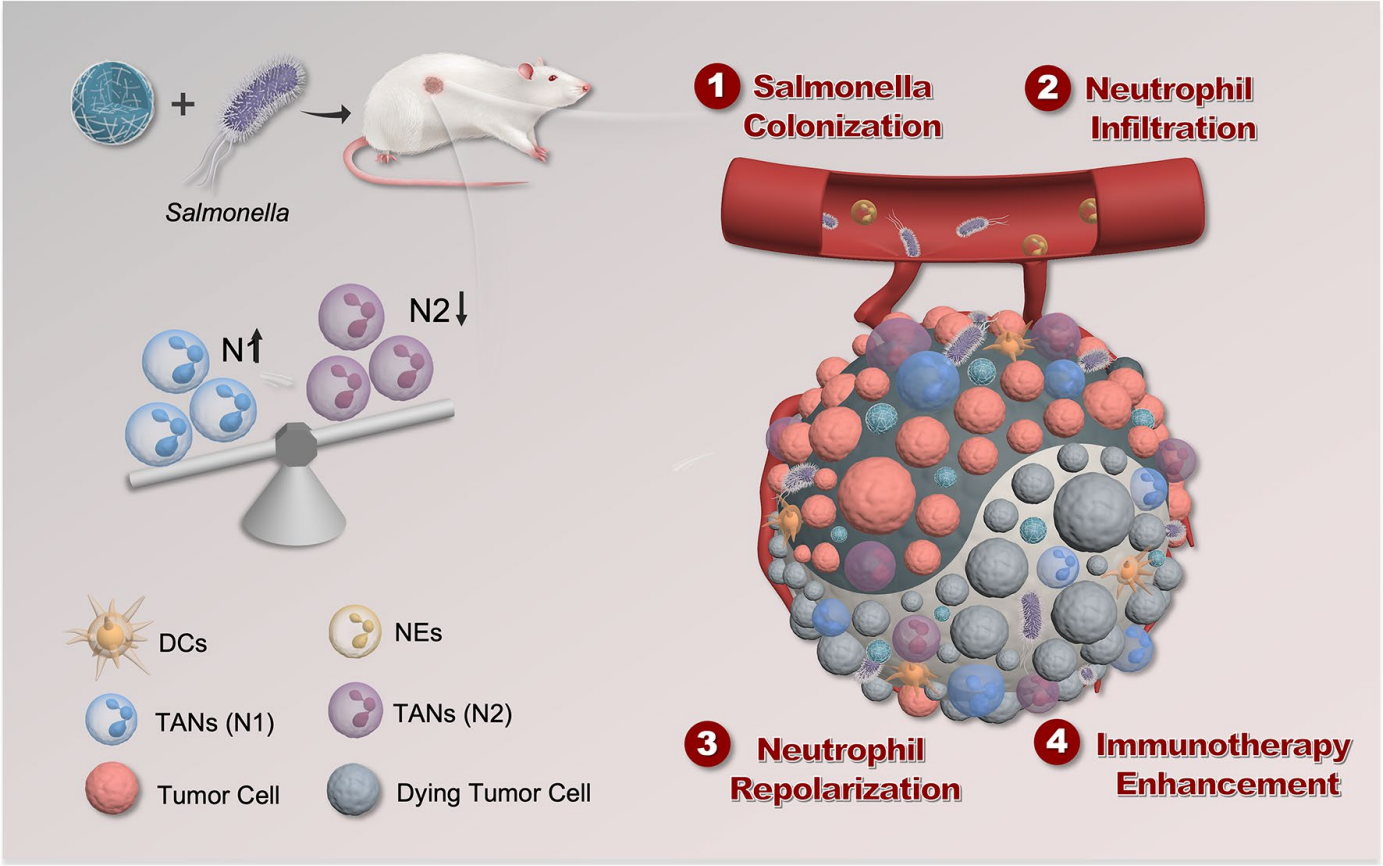

**Supplementary Information:**

The online version contains supplementary material available at 10.1186/s12951-024-02726-8.

## Introduction

Recent advances in immunotherapy and cell biology have introduced transformative cellular immunotherapy technologies into cancer treatment, providing promising prospects for individuals with malignant tumors [1]. Cellular therapy constitutes two categories based on cell type: innate immune cells and adaptive immune cells. Innate immunity encompasses innate immune cells, including macrophages, neutrophils, natural killer cells, and dendritic cells, among others, and these comprise the natural immunity responsible for swift and non-specific tumor recognition and elimination [2]. On the other hand, adaptive immunity, which is predominantly orchestrated by T cells, facilitates clearance mechanisms that are more targeted, impeding tumor recurrence [3]. Notably, T cell and dendritic cell therapies have achieved clinical success [4], and ongoing clinical trials explore other immune cell therapies via the use of NK cells and macrophages [5]. However, despite their pivotal role in innate immunity, there is still few research into immune therapy strategies rooted in neutrophils, possibly owing to their limited lifespan of merely 12–24 h [6]. Nonetheless, given their critical involvement in innate immunity, the potential of neutrophils in tumor treatment remains a promising avenue of research. Delving into tumor cell immunotherapy based on neutrophils holds considerable clinical significance.

Neutrophils are produced in the bone marrow and are primarily associated with inflammatory immunity [7]. Due to their high abundance and rapid response characteristics in the blood and their synergistic effects with other immune cells, neutrophils possess multiple potential advantages in terms of tumor treatment [8], and they can directly attack tumor cells and activate other immune cells by producing anti-tumor cytokines [9]. Current research mainly focuses on utilizing neutrophils as carriers to deliver targeted drugs to tumors. For instance, by inducing localized inflammation in tumor tissue through radiation therapy or surgical treatment and promoting neutrophil chemotaxis, drugs can be loaded on or anchored to neutrophils to enhance the accumulation of the drug in tumor tissue [10].

Similarly, our research team utilizes the facultative anaerobic property and immunogenicity of *Salmonella* (Sal) to construct a microbial immune microenvironment within local tumors, which recruits a large number of neutrophils to this site [11–14]. Capitalizing on this approach, researchers have used several delivery strategies to transform neutrophils into “Trojan horses”, achieving the targeted enrichment of anti-tumor drugs within tumors [15–17]. However, existing research mainly focuses on changes in the number of neutrophils at the tumor site without exploring the phenotype and functions of neutrophils in the tumor immune microenvironment [18]. A comprehensive understanding of the functions and roles of neutrophils in the tumor microenvironment can provide a basis for rational immune therapy methods [19].

**Scheme 1:**
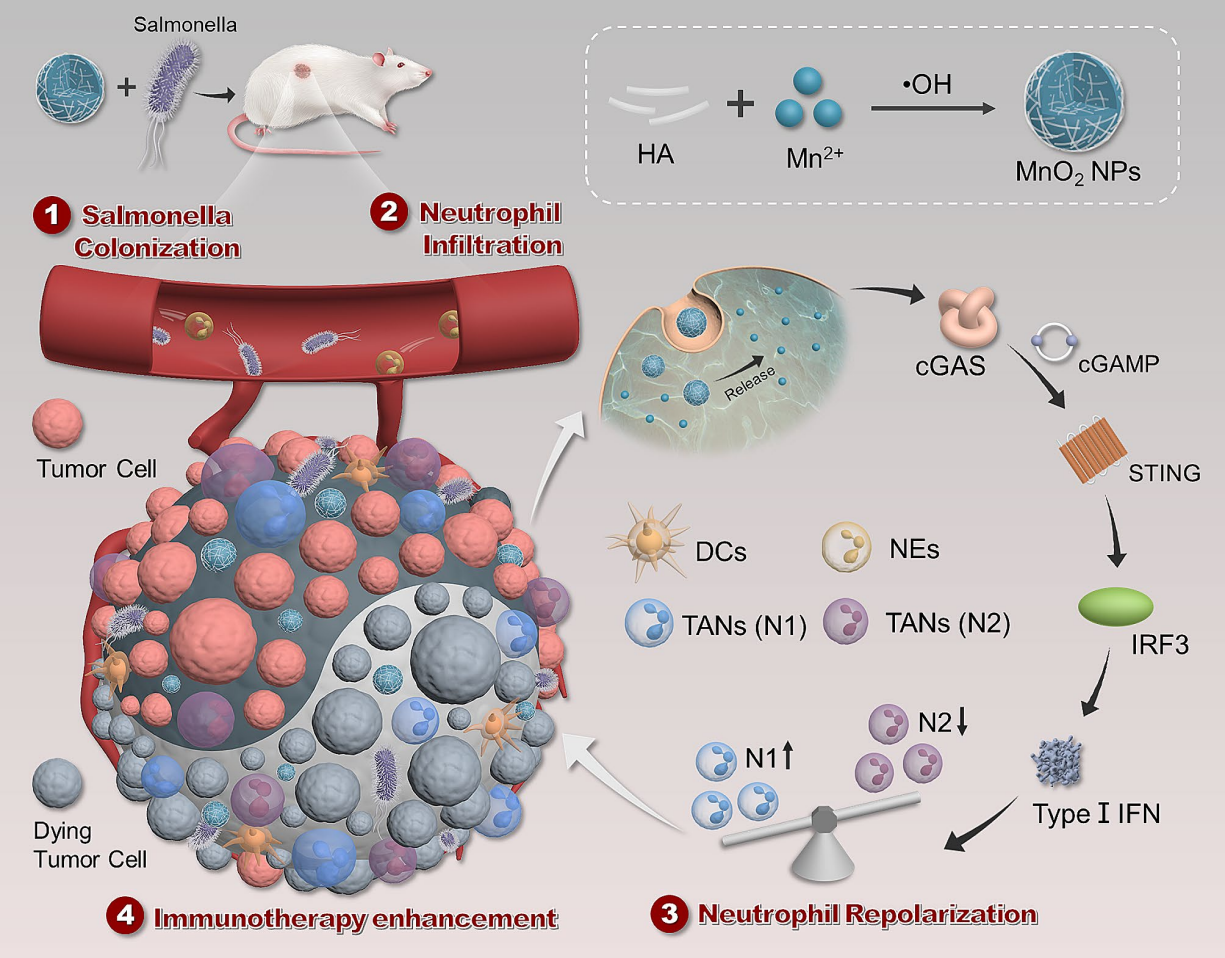
Schematic illustration of the preparation of MnO_2_ NPs and the synergistic tumor therapy of *salmonella* and MnO_2_ NPs. The combination therapy includes the following steps: (1) *Salmonella* colonizes in the tumor; (2) Neutrophils infiltrate into tumor recruited by the colonized *Salmonella*; (3) The Mn^2+^ released from MnO_2_ NPs activates the STING pathway to express type I IFN such as IFN-β, and then neutrophils get repolarized from the pro-tumor N2 phenotype to the anti-tumor N1 phenotype by these IFN-β; (4) These repolarized N1 neutrophils enhance the anti-tumor efficacy of *Salmonella* by transforming the tumor immune microenvironment

Neutrophils have a complex impact on the tumor immune microenvironment [8]. Similar to tumor-associated macrophages (TAMs), tumor-associated neutrophils (TANs) can exhibit either inhibitory or promotive effects regarding tumors, depending on their phenotypes, which are influenced by regulatory signaling molecules within the microenvironment [20]. For example, cytokines, such as IFN-β, IFN-γ, and TNF-α, promote polarization towards the anti-tumor N1 phenotype. In contrast, TGF-β and IL-10 tend to induce polarization towards the pro-tumor N2 phenotype [21]. In this work, we found that the neutrophils recruited by the presence of Sal, which then act on tumor tissue, primarily polarize towards the N2 phenotype, exerting a negative regulatory effect on microbial immune therapy. This suggests that the targeted regulation and repolarization of neutrophils represent a potential strategy to enhance the efficacy of Sal therapy. In order to achieve this, we developed manganese dioxide nanoparticles (MnO_2_ NPs) as an adjuvant for synergistic tumor therapy. We activated the STING signaling pathway in the tumor tissue using MnO_2_ NPs to promote the secretion of type I interferons [22–26], driving neutrophil repolarization toward the N1 phenotype (Scheme 1); this reshapes the tumor immune microenvironment, enhances the anti-tumor immune response, significantly improves the survival rate of tumor-bearing mice, and demonstrates good biological safety. Our study reveals the important in vivo limitations of microbial immune therapy and proposes solutions, providing effective strategies for enhancing microbial immunotherapy. Moreover, the study offers valuable insights into developing approaches for cell-based immunotherapy based on neutrophils 1.

## Results and discussion

### Tumor-specific colonization of engineered Sal and an assessment of therapeutic efficacy

**Fig. 1:**
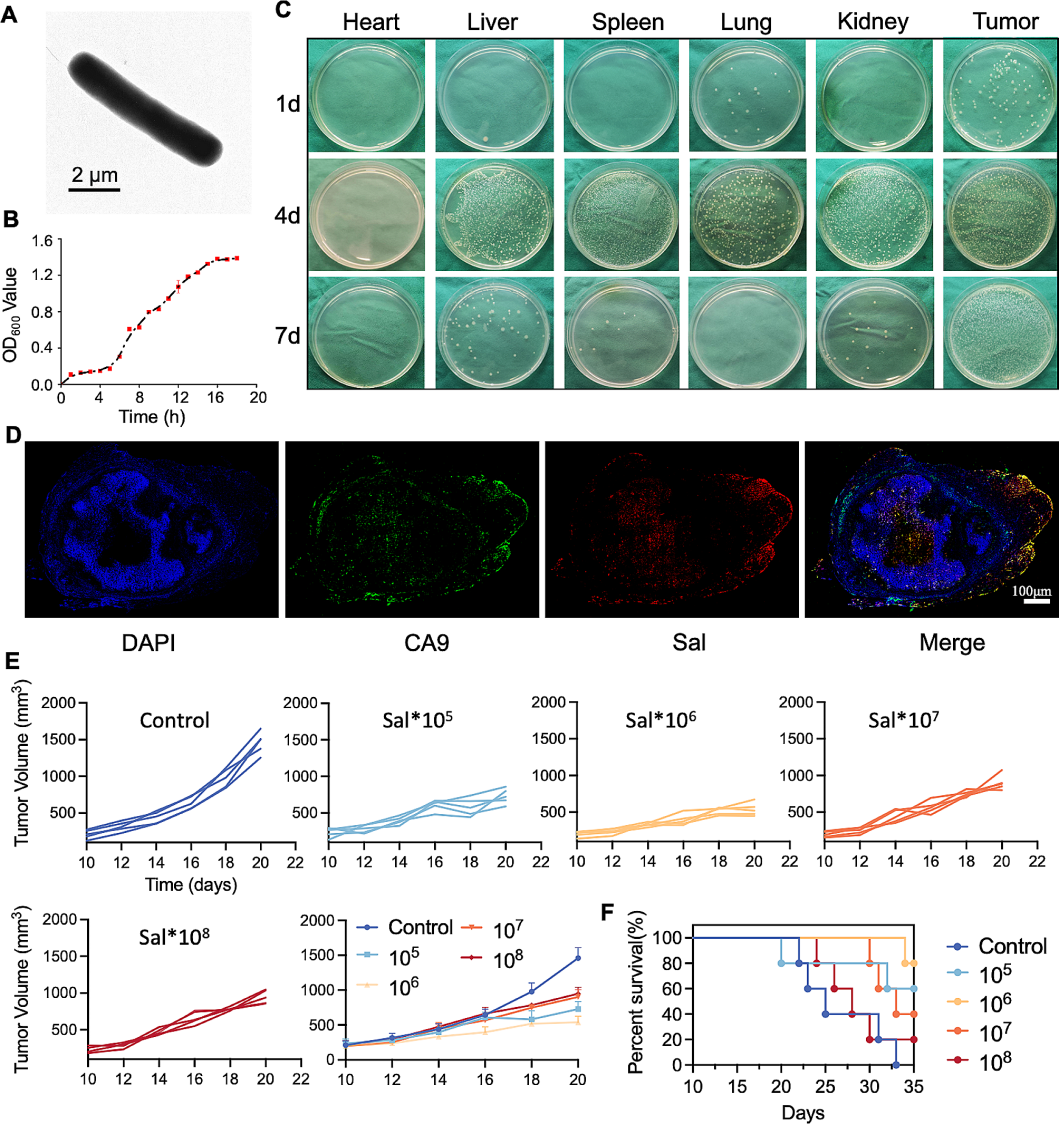
Characterization and antitumor activities of Sal. (**A**) TEM image of Sal. (**B**) The kinetics of bacterial growth represented by OD600 values. (**C**) The colonization of Sal in the major organs of mice for different preparations examined by the spread plate method (**D**) Immunofluorescence images of hypoxia and bacteria colocalization in tumor tissues at 48 h post Sal injection. (**E**) The tumor growth curves of mice post different treatments. (**F**) Survival rate of the mice with various treatments

The Sal used in this work was the engineered strain VNP20009, which the FDA has approved for clinical research. It has stable proliferative activity and favorable tumor-targeting properties. Transmission electron microscopy revealed the structure of the Sal as a blunt rod shape with an approximate length of 1–5 μm (Fig. 1A). Cultivation in an LB liquid medium exhibited dynamic bacterial growth, monitored by UV absorbance at 600 nm. Within 20 h, robust logarithmic growth was evident (Fig. 1B), indicating substantial proliferative activity.

As a facultative anaerobe, Sal exhibits a preference for colonizing tumor sites, but its non-specific distribution in normal tissue organs may cause side effects. Using a mouse 4T1 breast cancer xenograft model, we studied the in vivo distribution of the Sal after tail vein administration. We euthanized the mice and collected the organs (heart, liver, spleen, lung, kidney) and tumor tissues at various time points post-administration. After processing, we assessed colonization by plating the bacteria on LB agar. Notably, 1 day post-administration, the abundant presence of Sal in the tumor tissue significantly surpassed that of the normal organs (Fig. 1C, Figure S1), indicating preferential colonization and growth in the tumor microenvironment [27]. This can possibly be ascribed to the tumor microenvironment providing a more favorable environment for bacteria (such as hypoxia and a nutrient-rich, immunosuppressive microenvironment) [28–31]. Bacterial distribution was observed in multiple normal organs after 4 days of injections, suggesting non-specific bacterial diffusion and distribution in the mice. However, subsequent observations after 7 days revealed declining bacterial presence in the normal organs, which we attribute to innate immune clearance. The concentration remained high in the tumor tissue, showcasing long-term colonization and tumor-specific effects.

In order to corroborate the tumor-targeting abilities of Sal, we engineered it to emit red fluorescence, and we marked the hypoxic marker CA9 with green fluorescence in the hypoxic tumor areas for co-localization analysis [16]. Post intravenous injection, the fluorescence staining of the frozen tumor tissues indicated substantial overlap between the Sal and the hypoxic tumor regions (Fig. 1D), confirming its enrichment in deep hypoxic tumor areas due to its facultative anaerobic nature [16, 32]. These findings validate Sal’s ability to selectively target and colonize tumor regions while rapidly clearing from normal organs; this ensures safety for in vivo applications and establishes its viability for microbial immunotherapy in tumors.

With Sal’s targeting capabilities confirmed, we investigated its anti-tumor efficacy. The administration of varying doses of Sal into the 4T1-bearing mice and monitoring the subsequent tumor volume enabled a dynamic evaluation of therapeutic bacterial effects. Notably, different doses of Sal displayed significant tumor suppression compared to the control group (Fig. 1E). However, an intriguing observation emerged: the therapeutic effect did not exhibit a linear dose-dependent relationship. We enhanced tumor suppression efficiency by increasing the bacterial injection dose from 10^5^ to 10^6^ per mouse. However, beyond this point, further dose increments led to reduced efficacy. The mouse survival curve analysis echoed these findings (Fig. 1F). The dose-response pattern of bacterial therapy differs notably from conventional drugs, and one possible reason might be ascribed to the toxicity of the Sal at higher dosages. This necessitates further exploration of the underlying mechanisms to cultivate a more rational design for bacterial therapy doses and methodologies.

### Tumor-associated neutrophils as key regulators of Sal’s therapeutic efficacy

**Fig. 2:**
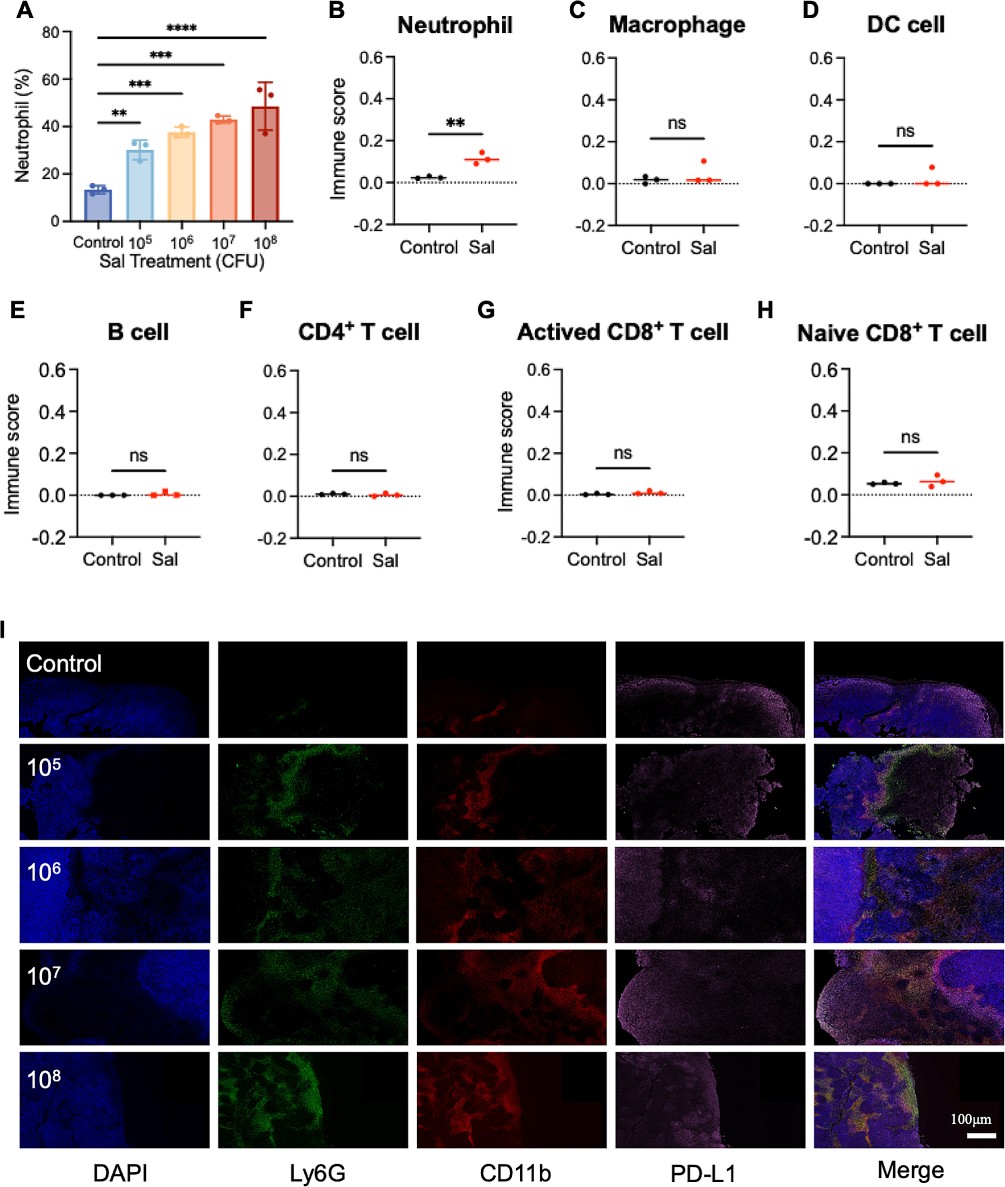
The effect on tumor-associated neutrophils after Sal’s therapeutics. (**A**) Quantity changes of neutrophils in the blood after different treatments. (**B-H**) The estimated immune subpopulations after different treatments. Statistical test of immune cell scores by Scatter plot (unpaired t test). (I) The tumor immunostaining of Ly6G, CD11b and PD-L1 post different treatments. Scale bar = 100 μm

While conventional drug therapies often exhibit dose-dependent trends within defined ranges, this behavior is not evident in the case of Sal. We hypothesize that this deviation might link to the intricate anti-tumor mechanisms of Sal and its influence on the tumor microenvironment. As a foreign microorganism, Sal can incite the body’s immune response against infection. Studies indicate that Sal colonization leads to the substantial recruitment of neutrophils to the tumor site [16]. Yet, the precise impact of neutrophil recruitment and activation on Sal’s therapeutic efficacy remains unclear. In order to explore this, we conducted routine blood tests on tumor-bearing mice (post-treatment) under various Sal doses, revealing a proportional increase in neutrophil content in the blood as the Sal dosage increased (Fig. 2A), indicating bacteria’s direct activating influence on neutrophils.

Activated neutrophils possess the ability to migrate towards sites of bacterial colonization, prompting alterations in the immune microenvironment. In order to probe this phenomenon, we performed mRNA transcriptome sequencing on Sal-enriched tumor tissue, coupled with immune cell proportion analysis using the CIBERSORT deconvolution method. Post-Sal treatment (Fig. 2B), we observed a notable rise in neutrophil presence within the tumor microenvironment. Conversely, other immune cell types (Fig. 2C–H), such as B cells, macrophages, DC cells, CD4^+^T cells, and CD8^+^T cells, showed no significant numerical discrepancies, implying that neutrophils act as the primary modulators of the Sal-colonized tumor immune microenvironment.

Previous research indicates the diverse effects of neutrophils on the tumor immune microenvironment, contingent upon their phenotypes [33]. Neutrophils can predominantly differentiate into either the anti-tumor N1 phenotype or the pro-tumor N2 phenotype. In order to explore neutrophil phenotypic characteristics, we conducted immunofluorescence staining by employing three markers—Ly6G (green fluorescence), CD11b (red fluorescence), and PD-L1 (pink fluorescence)—in the treated tumor tissue Sect. [34]. Accompanied by the increase in Sal dose, we noticed a substantial increase in the red/green co-localization signal indicative of neutrophils (Fig. 2I), which is consistent with the mRNA transcriptome sequencing outcomes mentioned earlier. Moreover, the co-localization of PD-L1 with neutrophils exhibited a notable increase, suggesting that neutrophils might predominantly adopt the N2 phenotype. This observation suggests that although higher doses of Sal can recruit more neutrophils, these cells primarily assume the N2 phenotype within the tumor microenvironment, potentially compromising the anti-tumor effect. Consequently, modulating the neutrophil phenotype emerges as a pivotal strategy for augmenting Sal’s anti-tumor efficacy by transforming the tumor immune microenvironment.

### Biocompatible MnO_2_ NPs as nano-activators of the STING pathway to induce IFN-β expression

The prior investigation has highlighted the pivotal role of tumor-associated neutrophil phenotypes in influencing the efficacy of Sal in anti-tumor therapy. Central to this influence is the promotion of the N1 phenotype of neutrophils while mitigating polarization towards the N2 phenotype. In order to modulate neutrophil phenotypes effectively, we developed manganese dioxide nanoparticles (MnO_2_ NPs) for this study. The design of the nanoparticles aims to activate the STING pathway within the tumor microenvironment by releasing Mn^2+^ intracellularly, thereby inducing IFN-β secretion and fostering polarization of neutrophils towards the N1 phenotype.

**Fig. 3:**
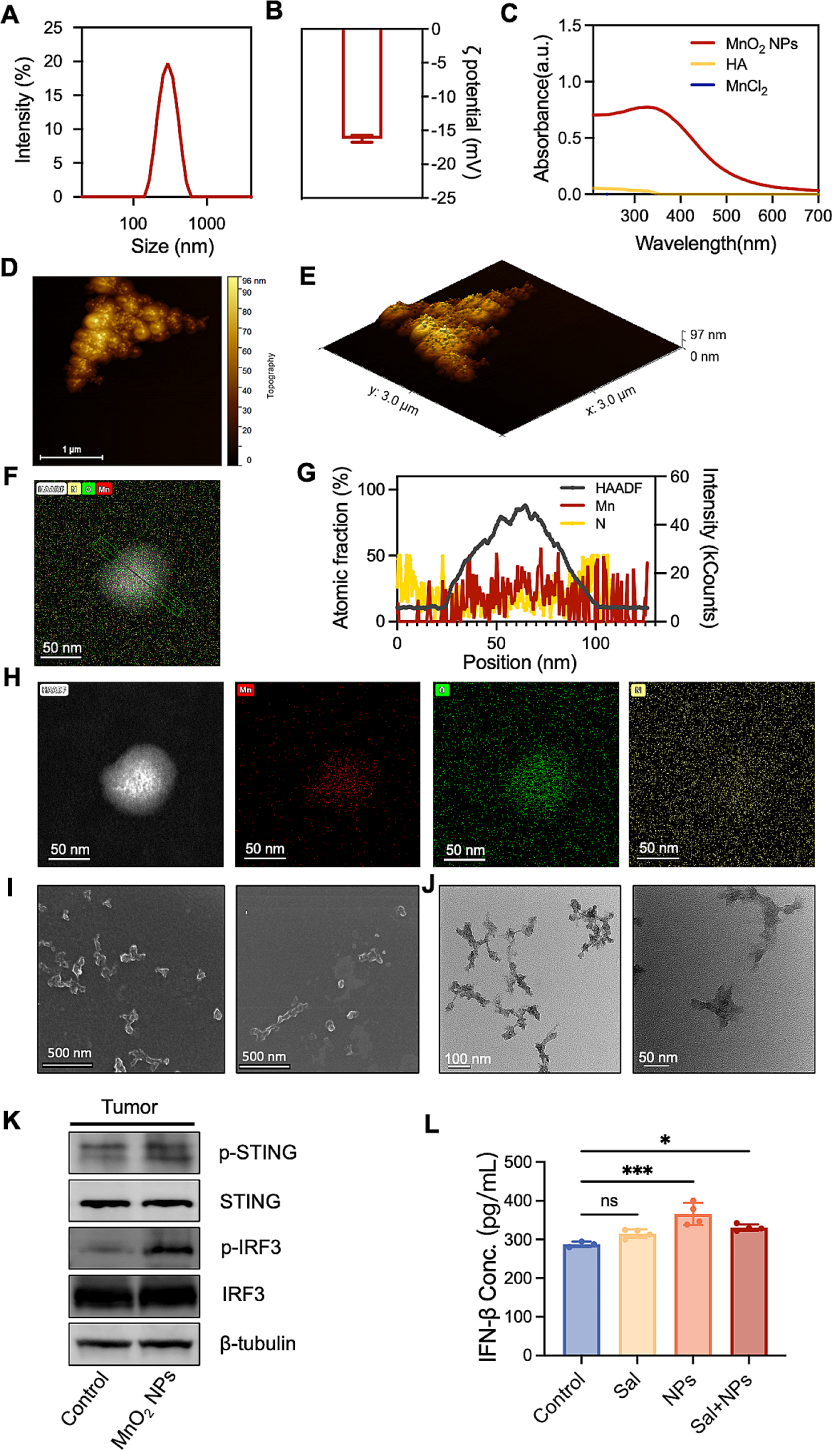
The characterization and STING pathway activating ability of MnO_2_ NPs. (**A**) The size distribution and (**B**) ζ potential of MnO_2_ NPs. (**C**) The UV-Vis spectra of HA, MnCl_2_ and MnO_2_ NPs. (D-E) The AFM microscopy of MnO_2_ NPs. (F-H) The elemental scanning and cross-sectional element statistics of MnO_2_ NPs. (I) TEM image and (J) SEM image of MnO_2_ NPs. (K) The protein expression of p-STING, STING, p-IRF3 and IRF3 in tumor tissues of mice post MnO_2_ NPs treatments detected by WB. (L) The level of IFN-β in the serum of mice post MnO_2_ NPs treatments detected by ELISA

The MnO_2_ NPs were synthesized by oxidizing Mn^2+^ under alkaline conditions and by employing hyaluronic acid (HA) as a template according to our previous work [35]. The hydrated particle size of the MnO_2_ NPs measured approximately 200 nm (Fig. 3A), with an ζ potential of roughly − 17 mV (Fig. 3B). The UV absorption spectra illustrated a characteristic shoulder peak within the 280–600 nm range, validating successful synthesis (Fig. 3C) [36]. AFM showed that the MnO_2_ NPs were assembled from multiple small spherical particles with dispersed adhesion to the clustered HA template, ensuring their stabilization (Fig. 3D and E). Elemental scanning and cross-sectional element statistics verified the distribution of the Mn, O, and N elements within the MnO_2_ NP structure (Fig. 3F,-H). The uniform dispersion of N elements within the particles further corroborated the stabilizing effect of the HA. The morphological analysis via SEM and TEM revealed that the prepared MnO_2_ NPs had irregularly aggregated spherical structures (Fig. 3I and J).

We conducted safety assessments of the prepared MnO_2_ NPs. The hemolysis rates at varying concentrations were below 5%, confirming the formulation’s safety for intravenous injection (Figure S2). Cytotoxicity studies revealed no significant inhibitory effects on the 4T1 tumor cells and DC cells within a specified concentration range (Figure S3), indicating the high biological safety of this nanoparticle formulation. The subsequent in vivo evaluations assessed the capability of the MnO_2_ NPs to activate the STING pathway post injection. The Western blotting analysis demonstrated a significant upregulation of phosphorylated STING protein (p-STING) and IRF3 protein (p-IRF3) in the tumor tissue of the mice following MnO_2_ NP treatment (Fig. 3K), confirming STING pathway activation by the MnO_2_ NPs. Additionally, ELISA assays revealed a substantial elevation in the IFN-β levels in the serum of the mice under the influence of the MnO_2_ NPs (Fig. 3L), affirming the capacity of the MnO_2_ NPs to stimulate IFN-β secretion by activating the STING pathway.

### In Vivo Regulatory Influence of MnO_2_NPs on Neutrophil Phenotype

**Fig. 4:**
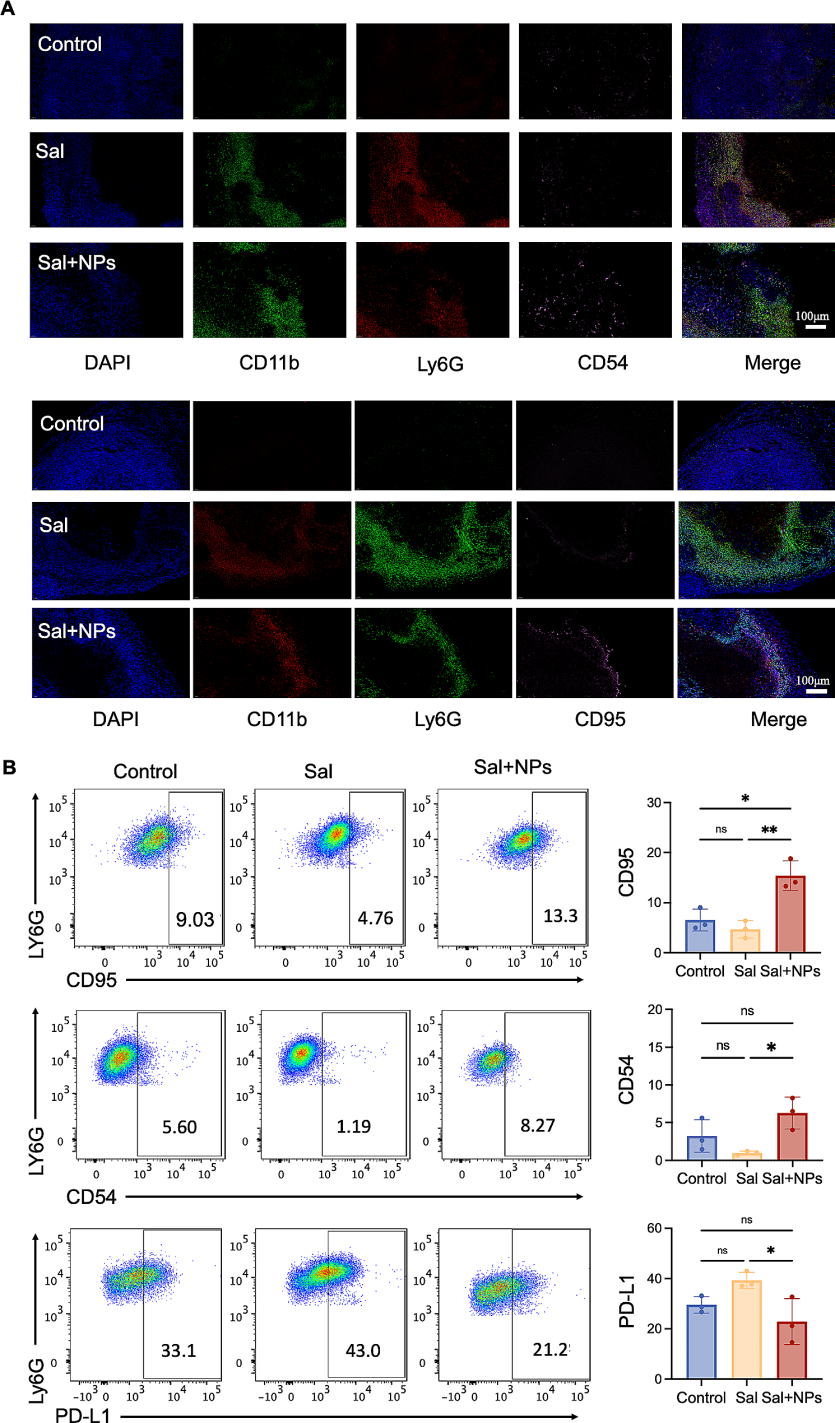
The regulatory influence of MnO_2_ NPs on neutrophil phenotype. (**A**) The immunofluorescence images of CD11b, Ly6G, CD54 and CD95 in tumor tissues of mice post different treatments. (**B**) The The expression of CD95, CD54 and PD-L1 by neutrophils detected by flow cytometry

Building upon the established role of MnO_2_ NPs in stimulating IFN-β secretion, we delved further into their potential to modulate the phenotypes of neutrophils. In order to assess this regulation, we conducted systematic staining of the surface markers of the tumor-associated neutrophils on the tumor tissue slices. The CD11b and Ly6G proteins served as markers for neutrophil localization, and CD54 (ICAM-1) and CD95 (Fas) were indicators of the N1 phenotype [37–40]. Our findings indicate a substantial increase in CD11b and Ly6G double-positive areas within the tumor region following Sal treatment (Fig. 4A), yet we observed no significant alteration in the CD54/CD95 fluorescence signals. However, treatment with Sal plus MnO_2_ NPs remarkably elevated the signals of CD11b, Ly6G, CD54, and CD95, suggesting the polarization of neutrophils towards the N1 phenotype upon their migration to the tumor tissue surface.

In order to corroborate the impact of combined Sal and MnO_2_ NP treatment on tumor-associated neutrophils, we performed single-cell isolation from the tumor tissue and used flow cytometry to ascertain their phenotype based on surface markers. The results post-Sal treatment indicated a notable reduction in the proportion of neutrophils exhibiting the CD95/CD54 phenotype (Fig. 4B), indicating a predominant shift towards the N2 phenotype. Concurrently, an increase in the proportion of PD-L1-positive cells signified an immunosuppressive tumor microenvironment [34]. However, upon combined treatment with Sal and the MnO_2_ NPs, there was a significant rise in the number of CD54/CD95-positive cells, coupled with a decrease in the number of PD-L1-positive cells, confirming that Sal and MnO_2_ NPs have complementary effects. We studied the dynamic change in neutrophil phenotype by co-staining CD54 and PD-L1. At 1 day post-Sal injection, neutrophils were polarized into the N2 phenotype. Following the local administration of MnO_2_ NPs, more neutrophils were repolarized into the N1 phenotype, which was maintained for the subsequent 4 days (Figure S4). Therefore, Sal facilitated neutrophil aggregation in the tumor area, and the MnO_2_ NPs modulated the phenotype of the neutrophils.

### Synergistic anti-tumor efficacy assessment and mechanistic exploration of Sal and MnO_2_ NPs combinatorial therapy

**Fig. 5:**
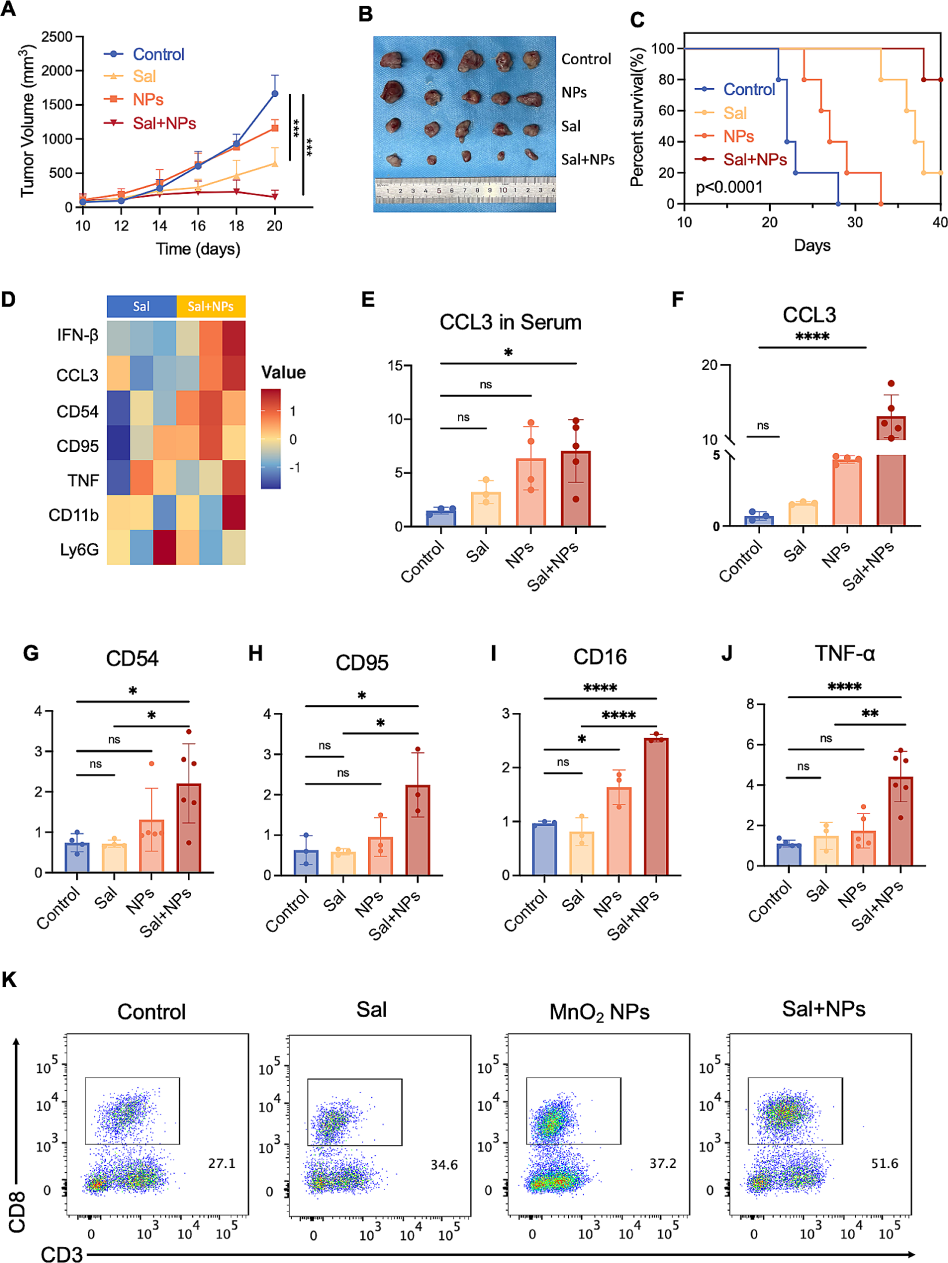
The synergistic anti-tumor efficacy and mechanistic Exploration of Sal and MnO_2_ NPs combinatorial therapy. (**A**) The tumor volume of mice and (**B**) their photographs of tumor tissues and (**C**) the survival rate of mice post different treatments. (**D**) Heat map of genes related to neutrophil phenotype according to the Database. (**E**) The level of CCL3 in serum detected by ELISA and (F-K) the level of CCL3, CD54, CD95, CD16, TNF-α in tumor tissues post different treatments detected by q-PCR. (L) The number of CD8^+^T cells in tumor post different treatments detected by flow cytometry

Subsequently, we investigated the synergistic impact of Sal and the MnO_2_ NPs on tumor treatment at the animal level. We dynamically monitored therapeutic efficacy throughout the treatment by recording mouse tumor volume (Fig. 5A). Notably, the MnO_2_ NPs exhibited modest anti-tumor activity compared to the control group, which we attribute to the potential immune-regulatory effect mediated by manganese [41]. Conversely, Sal demonstrated substantial tumor inhibitory effects; however, we observed tumor regrowth after 14 days. Among all the treatment groups, Sal plus MnO_2_ NPs displayed the most pronounced anti-tumor effect, completely arresting tumor growth within 20 days. The tumor tissues were collected post-treatment for direct observation, and the same results were obtained (Fig. 5B). According to the histological examination via H&E staining, we observed a notable reduction in the cell nuclei count and increased cell necrosis in the Sal plus MnO_2_ NPs treatment group (Figure S5). This signifies excellent anti-tumor efficacy. Survival curve assessment further validated the optimal therapeutic effect of Sal plus MnO_2_ NPs, achieving an 80% survival rate within 40 days (Fig. 5C).

Our subsequent exploration aimed to elucidate the synergistic mechanism of Sal and MnO_2_ NPs. The mRNA transcriptome sequencing heatmaps depicted significant alterations in gene expression upon adding MnO_2_ NPs to the Sal treatment group (Figure S6). The neutrophil-related genes were screened for enrichment, revealing a substantial increase in CCL3 gene expression for the Sal treatment groups (Fig. 5D). This indicates that the chemokines stimulated neutrophil enrichment in the tumor tissue. Besides, measurements of the CCL3 levels in the plasma and tumor tissue post-Sal treatment exhibited significant upregulation (Fig. 5E and F), validating the aforementioned mRNA results. It should be noted that CCL3 plays a pivotal role in tumor immunotherapy. CCL3 acts as a chemokine that not only recruits neutrophils and promotes Th1 immune responses but also enhances the recruitment and activation of CD8 + T cells. These mechanisms collectively contribute to a robust anti-tumor immune response, underscoring the therapeutic potential of nanoparticle-induced CCL3 upregulation.

The sequencing outcomes also indicated a significant elevation in the mRNA levels of CD95 and CD54 following Sal combined with MnO_2_ NP treatment when compared to Sal treatment alone. Similarly, the experimental measurements displayed a marked increase in those markers pertinent to the anti-tumor neutrophils, such as CD54, CD95, and CD16 (Fig. 5G-I), affirming the N1-oriented polarization effect of neutrophils under the influence of MnO_2_ NPs [34]. This can be explained by the significant elevation of IFN-β gene value which also validates the aforementioned STING pathway activating results. Correspondingly, the sequencing and content determination confirmed a substantial rise in TNF levels (Fig. 5J), signifying enhanced activation of anti-tumor immunity. The increased number of infiltrated CD8^+^ T cells detected via flow cytometry within the mice tumors corroborates this effect (Fig. 5K). In summary, the synergistic enhancement mechanism of Sal and MnO_2_ NPs is attributed to their regulatory impact on neutrophils, enhancing anti-tumor immune effects and microbial immunity.

Finally, we evaluated the biosafety of this treatment strategy at the animal level. There were no significant changes in the weight of the mice across all treatment groups throughout the treatment period (Figure S7). The post-treatment serum analysis of the biochemical indicators, including ALT, AST, CRE, and BUN, showed that normal ranges were maintained (Figure S8), indicating no liver or kidney toxicity. Further histological analysis of the major organs in the mice via H&E staining (Figure S9) revealed no significant pathological changes post-treatment, highlighting good biosafety.

## Conclusion

In conclusion, this study illuminates the critical but relatively unexplored role of neutrophils in bacteria-mediated tumor therapy, and introduces a therapeutic strategy amalgamating Sal with MnO_2_ NPs to synergistically enhance efficacy. While Sal’s colonization within tumors recruits a substantial neutrophil cohort, these neutrophils predominantly polarized into N2 phenotype, contributing to the establishment of an immunosuppressive microenvironment that curtails the effectiveness of microbial immunotherapy. To surmount this challenge, MnO_2_ NPs were engineered to activate the STING pathway, instigating the secretion of interferons and orchestrating the repolarization of neutrophils towards an anti-tumor N1 phenotype. This strategic reprogramming heralds a paradigm shift in the tumor immune microenvironment. Experimental outcomes underscore the potency of this combined treatment strategy in effectively impeding tumor growth, enhancing mouse survival rates, and demonstrating favorable biological safety profiles. Our findings engender a nuanced comprehension of neutrophil dynamics in tumor therapy, offering a promising avenue to fortify the effectiveness of microbial immunotherapy. This comprehensive inquiry not only proffers resolutions to the challenges encountered in microbial immune therapy, but also lays a robust groundwork for prospective advancements in cell-based immunotherapy, centered on harnessing the potential of neutrophils for refined cancer treatment strategies.

## Materials and methods

### Materials

MnCl_2_ and NaOH were purchased from Aladdin (Shanghai, China). 1640 Medium, fetal bovine serum (FBS) and penicillin-streptomycin solution were purchased from Gibco Life Technologies, Inc. (Grand Island, NY, USA). 4’,6-diamidino-2- phenylindole (DAPI) and LB broth were purchased from Solarbio Biotech Co., Ltd. (Beijing, China), and 3-(4,5-dimethylthiazol-2-yl)-2,5-diphenyl tetrazolium bromide (MTT) was from Sigma-Aldrich Co., Ltd. (St. Louis, MO, USA). Protein phosphatase inhibitor (K1015) was obtained from APExBIO (Houston, USA). Rabbit mAb p-STING (19,781) and Rabbit mAb p-IRF3 (4947) were purchased from Cell Signaling Technology, Inc. (Massachusetts, USA). Rabbit pAb STING (A20175) and Rabbit mAb IRF3 (A19717) were purchased from Abclonal, Inc. (Wuhan, China). Mouse monoclonal antibody to Ly6G (127,628, 127,613), CD3(100,234), CD8(100,733), CD11b (101,215, 101,261), CD95(152,607) and CD54(116,105) were obtained from Biolend, Inc. (California, USA); antibody to CD45(561,037) and PD-L1(568,315) were obtained from Becton Dickinson Company (BD, New Jersey, USA). ELISA Assay Kit of IFN-β (JL20219) and CCL3 (JL20414) were obtained from JiangLai Biology (Shanghai, China). ELISA Assay Kit of IFN-β and CCL3 were obtained from JiangLai Biology (Shanghai, China).

### Tumor cell line and bacteria

We obtained the 4T1 breast cancer cell line from the cell bank of the Chinese Academy of Sciences (Shanghai, China). The cells were cultured at 37 °C and 5% CO_2_ in a 1640 medium supplemented with 10% FBS and 1% penicillin-streptomycin solution. Kai Yang (Suzhou University, China) kindly provided the plasmids and bacterial strains (*Salmonella*, VNP20009) used in this study. The bacteria were grown at 37 °C in LB broth with shaking at 180 rpm.

### Animals

Healthy female BALB/c mice (6–8 weeks) were purchased from the Department of Laboratory Animals, Central South University (Changsha, China), and were housed in environmentally controlled conditions (22 °C and a 12 h light/dark cycle) with ad libitum access to standard laboratory chow and water. The local Institutional Ethics Review Committee approved the study protocol, and the animal studies were carried out in accordance with the established ethical guidelines for animal use and care at Central South University. The 4T1 tumor-bearing BALB/c mouse model was prepared by subcutaneously placing 4T1 cell suspension (5 × 10^7^ pieces/mL; 100 µL/mouse) on the right side of the back of the mice.

### Characterization of sal

Morphological observation protocol: Take an appropriate amount of Sal and centrifuge at 3000 rpm for 5 min. Collect the precipitate, resuspend it, drop it onto the surface of the copper mesh, and then dry it in an electric hot air drying oven. Repeat this process four times and observe the morphology of the sample using TEM.

Growth curve of Sal in vitro: The attenuated Sal strain VNP20009 was grown at 37 °C overnight in a fresh liquid LB medium to reach an optical density (OD_600_) of 1. Then, the medium was diluted 10 times by fresh liquid LB and cultured at 37 °C for 24 h. The OD600 values were measured at predetermined time points, and a bacterial growth curve was drawn.

### Colonization of sal in hypoxic tumor region

The 4T1 tumor-bearing BALB/c mice were intravenously injected with sterile saline containing 6 × 10^6^ CFU of Sal. The tumor tissues were harvested 48 h post-injection, and they were placed on the cryostat for quick freezing. This was followed by embedding them to obtain the frozen slices. The slices were incubated with primary antibodies against carbonic anhydrase-IX (CA9) at 4 °C overnight; they were then incubated with fluorescent-labeled secondary antibodies for 10 min. After being counterstained with DAPI, the fluorescence images were obtained by CLSM.

### Therapeutic effect of Sal in different concentrations

The 4T1 cells suspended in PBS were inoculated subcutaneously on the back of the BALB/c mice (5 × 10^7^ per animal). When the tumor volume reached 500 mm^3^, Sal at different doses (10^5^~10^8^ CFU/mice) was injected intravenously into the mice. The tumor volume was calculated using the following equation: tumor volume = length × (width)^2^/2. The number of dead mice was recorded every day, and the survival curve was drawn. The mice were assumed to be dead when the tumor volume exceeded 2000 mm^3^ or physiological death occurred.

### Neutrophil ratio and recruitment in peripheral blood

We injected different concentrations of Sal into the tail vein of the Balb/c tumor-bearing mice, with the dose calculated as 1 Sal × 10^5^, 1 × 10^6^, 1 × 10^7^, 1 × 10^8^CFU/piece. For the neutrophil ratio, after 24 h, the blood of the mice was collected and placed in an anti-coagulant tube for routine blood examination, and the proportion of neutrophils was recorded. For the neutrophil recruitment measurement, after 24 h, the tumors were collected under cryopreservation. We performed mRNA transcriptome sequencing on the Sal-enriched tumor tissue, coupled with immune cell proportion analysis using the CIBERSORT deconvolution method.

### Preparation and characterization of MnO_2_ NPs

Using HA as a template, the MnO_2_ NPs were prepared by the oxidation of Mn^2+^ under alkaline conditions. We used a particle size analyzer, SEM, TEM, and AFL to sequentially analyze the particle size distribution, the ζ characterization of potential, the elements, and morphology of the MnO_2_ NPs.

### Western blot for STING pathway

We cut approximately 100 mg of mouse tumor and placed it into a homogenization tube, added RIPA lysis solution (containing 1% PMSF and 1% phosphatase inhibitor) in a ratio of 100 mg-to-1 mL, homogenized this at 65 Hz for 160 s in an homogenizer, and then lysed it at 4 °C for 1 h. Then, we centrifuged the sample at 13,000 rpm for 15 min and collected the supernatant as the total protein sample. We used a WB visualizer to detect the expression of immune-related proteins: STING, p-STING, IRF3, and p-IRF3.

### Immunofluorescence for neutrophils

After dewaxing the mouse tumor paraffin sections in xylene, they were subjected to gradient hydration with 100%, 95%, 85%, and 75% ethanol, as well as distilled water, followed by antigen repair. We added normal goat serum and incubated the samples at room temperature for 60 min for sealing. After completion, the primary antibodies (CD11b, Ly6G, CD95, CD54, and PD-L1), secondary antibodies, and DAPI stainings were incubated separately. Finally, an appropriate amount of anti-fluorescent quenching sealing solution was added to the center of the tissue slice, covered with a cover glass; this was imaged under a fluorescence microscope.

### Flow cytometry for neutrophils

The fresh mice tumors were taken and cut into small pieces, then ground on a cell filter and rinsed repeatedly with PBS. Single-cell suspension was collected from the well and centrifuged for 5 min. An appropriate amount of red blood cell lysis solution was added to the cell precipitate. After 2 min of lysis, the samples were centrifuged at 1000 rpm for 5 min. The upper layer of red, clear solution was discarded, and the sample was washed once with PBS. To each tube, 200 µL of CD16/32 antibody (1:100) was added and incubated at 4 °C for 10 min. Fluorescently labeled antibodies (CD45, CD3, CD4, CD8, CD11b, and Ly6G) were then added to each sample tube, and blank tubes and single staining tubes were set up for flow cytometry detection. The proportion of various cells in the tumor tissue was analyzed using FlowJo software.

### Tissue staining H&E

The collected tumor tissue was fixed with 4% paraformaldehyde for 24 h and then embedded in paraffin and sliced. The slices were stained with H&E staining, and the images were obtained through an optical microscope. We collected the major organs, including the heart, liver, spleen, lungs, and kidneys, at the end of the experiment and performed H&E staining, as described above.

### Anti-tumor activity In Vivo

The 4T1 cells suspended in PBS were inoculated subcutaneously on the back of BALB/c mice (5 × 10^7^ per animal). When the tumor volume reached ~ 500 mm^3^, the mice were divided into four groups (six mice per group). Groups 1 and 3 were injected intravenously with PBS, whereas Groups 2 and 4 were injected intravenously with Sal at a dose of 1 × 10^6^ CFUs per mouse. After 2 days, Groups 3 and 4 were injected intratumorally with MnO_2_ NPs (containing Mn 5 mg/kg) and Groups 1 and 2 with PBS. This injection was repeated twice. The body weight and tumor volume were recorded every day for 15 days. Tumor volume was calculated according to the formula given above. On the 16th day, the mice were euthanized. The tumors and main organs from the mice of the different groups were harvested and photographed. The number of dead mice was recorded every day, and the survival curve was drawn. The mice were assumed to be dead when the tumor volume exceeded 2000 mm^3^ or physiological death occurred.

### Blood biochemistry analysis

The 4T1 tumor-bearing BALB/c mice were treated as the procedure above. The blood samples were taken 48 h post-injection. The levels of aspartateaminotransferase (AST), alanine aminotransferase (ALT), blood urea nitrogen (BUN), and creatinine (CRE) were determined using a Biochemical Auto Analyzer.

### Electronic supplementary material

Below is the link to the electronic supplementary material.

Supplementary Material 1

## Data Availability

No datasets were generated or analysed during the current study.
