# Supplementary material for: Repolarizing neutrophils via MnO2 nanoparticle-activated STING pathway enhances Salmonella-mediated tumor immunotherapy
Source: J Nanobiotechnology. 2024 Jul 27;22:443. doi: 10.1186/s12951-024-02726-8 (PMC11282601; doi:10.1186/s12951-024-02726-8)
Supplement: Supplementary file 1 — Supplementary Material 1 [file 12951_2024_2726_MOESM1_ESM.docx]

**Figure S1.** The bacterial load quantification in major organs.


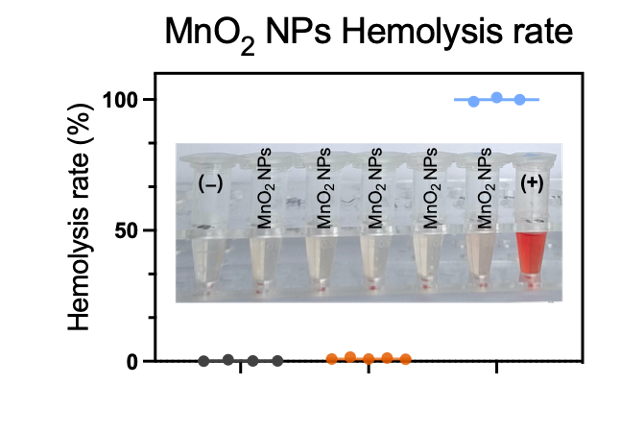


**Figure S2.** The hemolysis rates at varying concentrations of MnO_2_ NPs from 10, 20, 50, 100, 200 μg/mL.


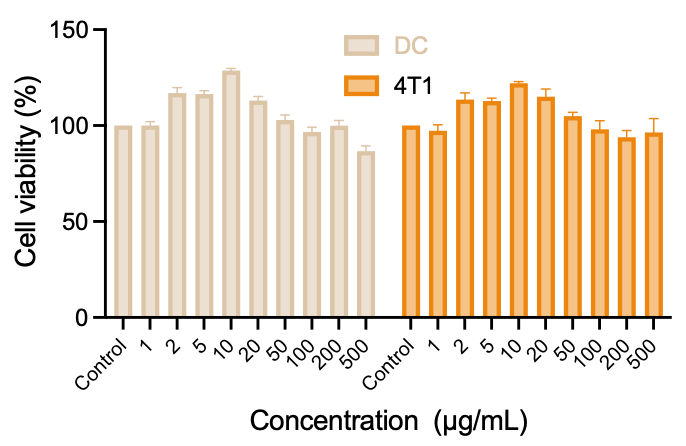


**Figure S3.** The Cell viability of DC and 4T1 cells after incubating with different concentrations of MnO_2_ NPs for 48 h.


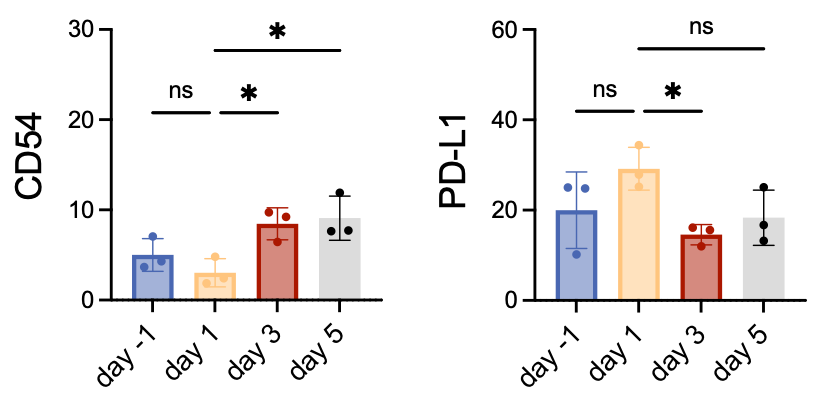


**Figure S4.** The expression of CD54 and PD-L1 by neutrophils detected by flow cytometry.


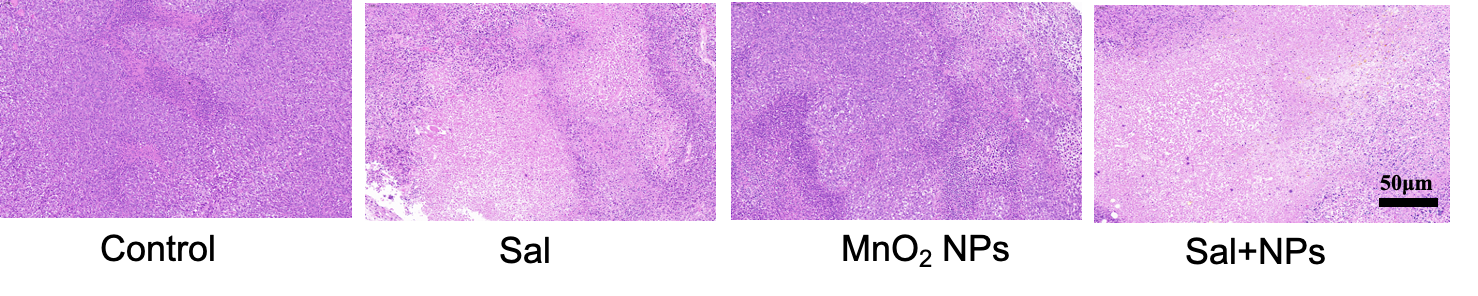


**Figure S5.** The H&E staining of tumor post different treatments.


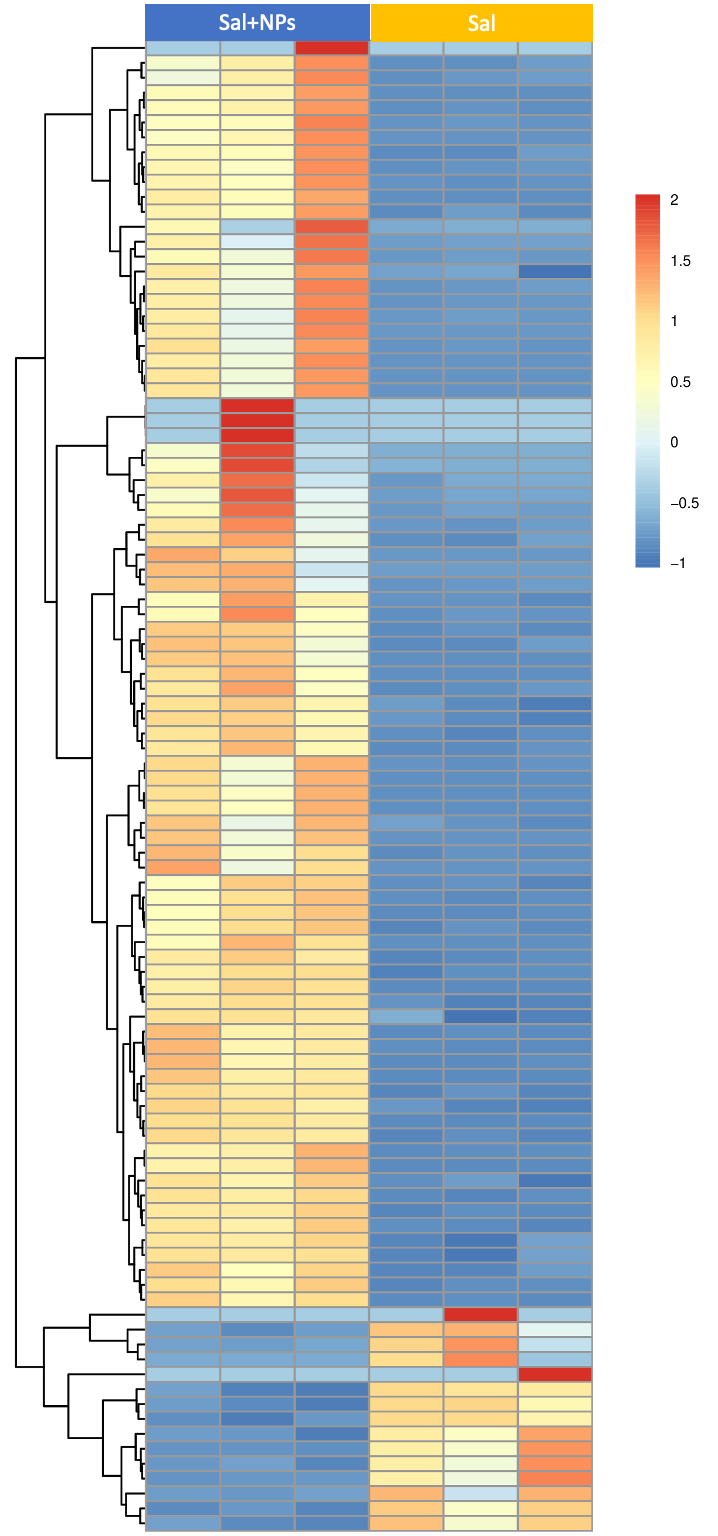


**Figure S6.** (A) The mRNA transcriptome sequencing heatmaps of tumor tissue post different treatments.


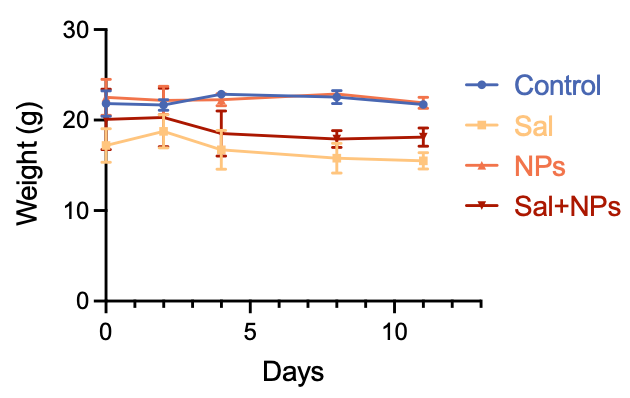


**Figure S7.** The weight curve of the mice across all treatment groups.


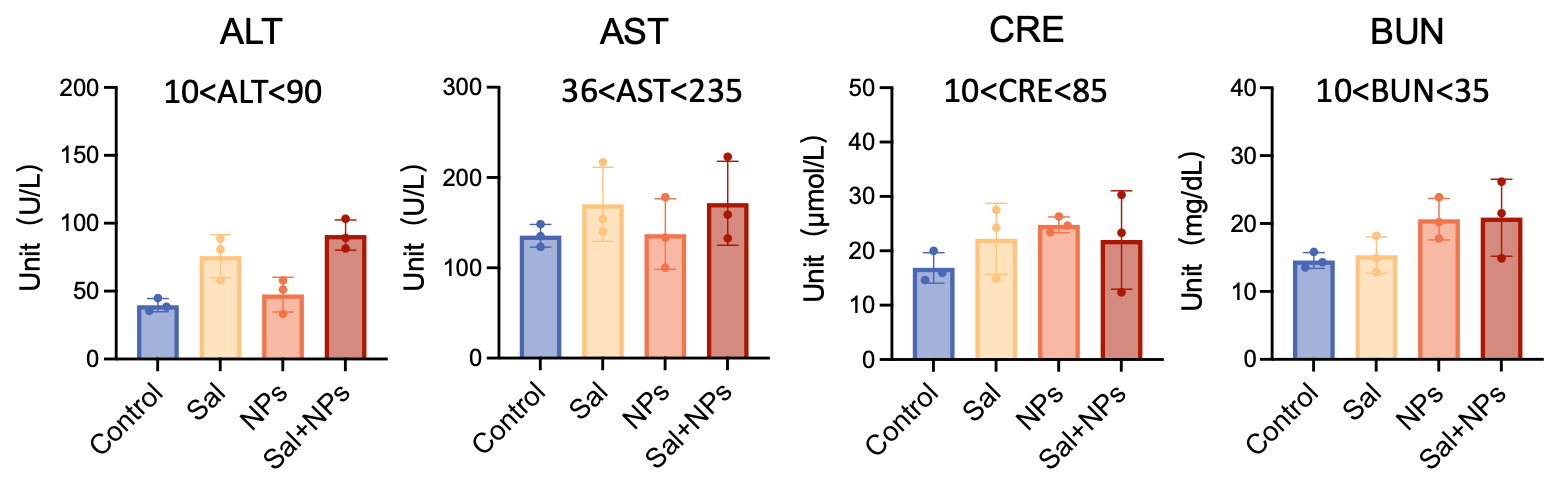


**Figure S8.** The post-treatment serum analysis of the biochemical indicators.


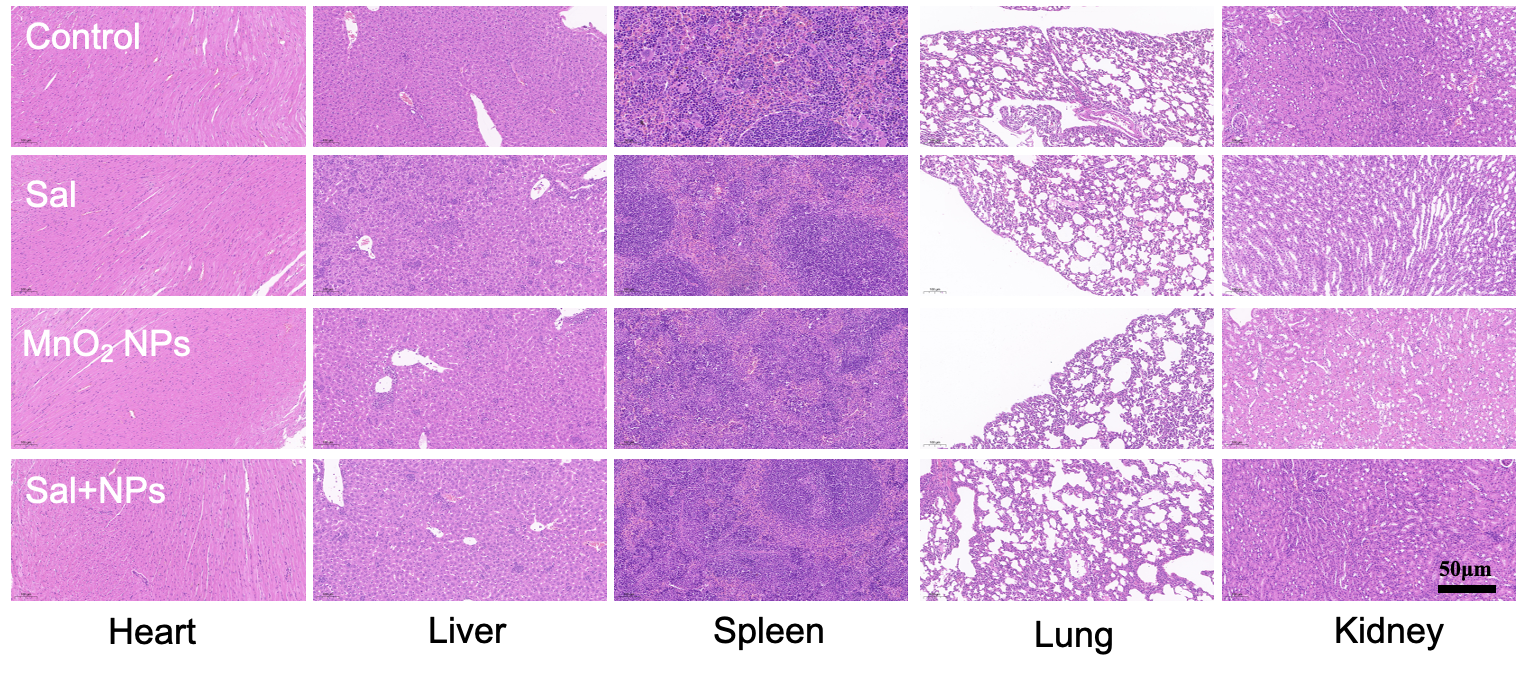


**Figure S9.** The H&E staining analysis of the major organs in the mice.
